# A mechanistic review of chinese medicine polyphenols on bone formation and resorption

**DOI:** 10.3389/fphar.2022.1017538

**Published:** 2022-10-12

**Authors:** Yan Li, Lingyu Li, Xiaoyun Li, Bingjie Luo, Qianyun Ye, Haoyu Wang, Li Yang, Xiaofeng Zhu, Li Han, Ronghua Zhang, Huaqin Tian, Panpan Wang

**Affiliations:** ^1^ College of Traditional Chinese Medicine, Jinan University, Guangzhou, China; ^2^ Cancer Research Institute, Jinan University, Guangzhou, China; ^3^ Guangdong Provincial Key Laboratory of Traditional Chinese Medicine Informatization, Jinan University, Guangzhou, China; ^4^ College of Pharmacy, Jinan University, Guangzhou, China; ^5^ First Affiliated Hospital of Jinan University, Guangzhou, China; ^6^ Foshan Hospital of Traditional Chinese Medicine, Foshan, China

**Keywords:** Chinese herbal medicine, polyphenols, bone resorption, bone formation, osteoblast, osteoclast

## Abstract

Bone reconstruction includes a steady state system of bone formation and bone absorption. This tight coupling requires subtle coordination between osteoblasts and osteoclasts. If this balance is broken, it will lead to bone mass loss, bone density reduction, and bone metabolic diseases, such as osteoporosis. Polyphenols in Chinese herbal medicines are active ingredients in plant extracts with high safety and few side effects, and they can play a role in affecting bone formation and bone resorption. Some of these have estrogen-like effects and can better target bone health in postmenopausal women. The purpose of this review is to provide comprehensive information on the mechanisms underlying the relationship between traditional Chinese medicine polyphenols and bone formation or bone resorption.

## 1 Introduction

Bones are essential to the human body, providing structural support, protecting vital organs such as the bone marrow and brain, promoting blood production, and serving as a reservoir of minerals. From birth to death, bones are constantly reshaped to maintain critical functions and maintain constant changes in bone mass.

Bone remodeling is achieved through the tight coupling of bone resorption and bone formation, and this is closely related to the participation of osteoclasts and osteoblasts. Bone marrow-derived osteoclasts are responsible for the absorption of aged bone, and mesenchymal osteoblasts are responsible for the synthesis and mineralization of new bone. If this balance is broken, such as increased bone resorption that is not compensated for by a similar increase in bone formation, this will lead to bone mass loss, bone density reduction, and bone metabolic diseases, such as osteoporosis (Crotti et al., 2015). Bone remodeling is regulated by multiple local cytokines (e.g., platelet-derived growth factor (PDGF), insulin-like growth factor [IGF), beta tumor growth factor (TGF)], and systemic hormones (growth hormones, parathyroid hormone (PTH), insulin, and oxytocin), vitamin d, energy metabolism (Karsenty and Khosla, 2022), and the regulation of multiple signaling pathways. Among these, Wnt, TGFβ, RANK/RANKL, and the M-CSF/C-FMS pathway regulate the differentiation and activity of osteoclasts. The Runt-associated transcription factor 2 (Runx 2), Osterix (Osx), β-catenin, activating transcription factor 4 (Atf 4), and the activating protein 1 (AP-1) family are the primary transcription factors involved in osteoblast differentiation (Chau et al., 2009; Soltanoff et al., 2009; Meyer et al., 2014). However, recent research suggests that positioned bone is also an important organ with paracrine and endocrine functions. Moreover, there is crosstalk between osteoblasts and osteoclasts that allow them to communicate and influence each other. The sympathetic nervous system (SNS) also has an effect on bone balance (Karsenty and Khosla, 2022).

For the past 3 decades, the mainstay of treatment for osteoporosis has been antiresorptive agents (e.g., bisphosphonates) that reduce fracture risk through continuous administration. However, some epidemiological studies have shown an association between long-term bisphosphonate therapy and atypical femoral fractures (AFF) (Shane et al., 2014). Therefore, these drugs are not suitable for long-term use for the treatment of bone-damaging diseases, and they may not be suitable as oral drugs either. In addition, long-term medication can cause problems such as gastrointestinal (GI) toxicity, weight loss, bone pain, low calcium levels (Lu et al., 2016; Gao et al., 2017; Grigg et al., 2017; Lange et al., 2017; Monda et al., 2017). Hence, potential new drugs are urgently needed to replace existing treatment strategies due to clinically adverse effects (Estell and Rosen, 2021).

Chinese herbal medicine has been used for many centuries. Polyphenols are the active ingredients extracted from Chinese herbal medicine. A polyphenol is a type of plant component that widely exists in plants and contains a variety of hydroxyphenols. They are important secondary metabolites in plants, with polyphenol structures. Polyphenols are primarily found in the bark, roots, shells, leaves, and fruits of plants. Polyphenols can be divided into flavonoids, phenolic acids, lignans, and stilbenes according to their structure. As bioactive molecules, polyphenols derived from Chinese herbal medicines have been shown to have many effects on human health by acting on different biological systems. Polyphenols have many physiological activities such as anti-osteoporosis, anti-oxidation, anti-infection, anti-tumor, and anti-atherosclerosis activities. In addition, a large number of studies have shown the effectiveness of polyphenols in the treatment of bone related diseases (Tao et al., 2016; Zou et al., 2016; Chen et al., 2017). Polyphenols can play a role in bone reconstruction by affecting bone formation and bone resorption. Polyphenols act on osteoclasts, osteoblasts and bone marrow mesenchymal stem cells, regulate several important signal pathways, and play a role in bone remodeling. In addition, these polyphenols are low cost and have fewer adverse reactions. Therefore, they are more suitable for long-term use than synthetic drugs. Hence, their therapeutic potential would represent a new approach for future drug discovery and development based on polyphenol extracts from Chinese herbal medicines.

**FIGURE 1:**
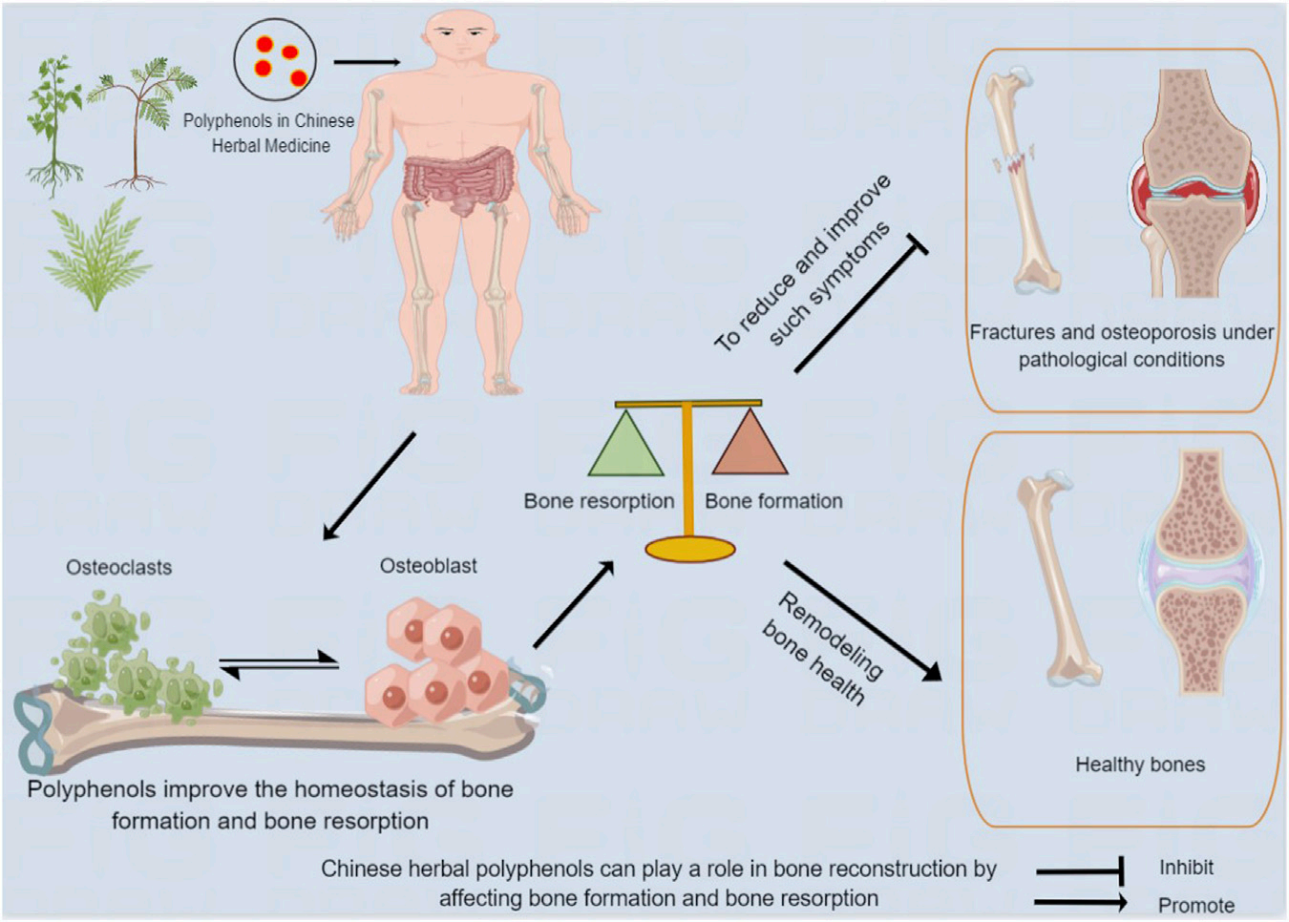
Chinese herbal polyphenols can play a role in bone reconstruction by affecting bone formation and bone resorption. After being absorbed in the gastrointestinal tract, Chinese medicine polyphenols act on human bones, and improve bone absorption and bone formation, contributing to bone remodeling and health, and reducing and improving adverse events.

**FIGURE 2:**
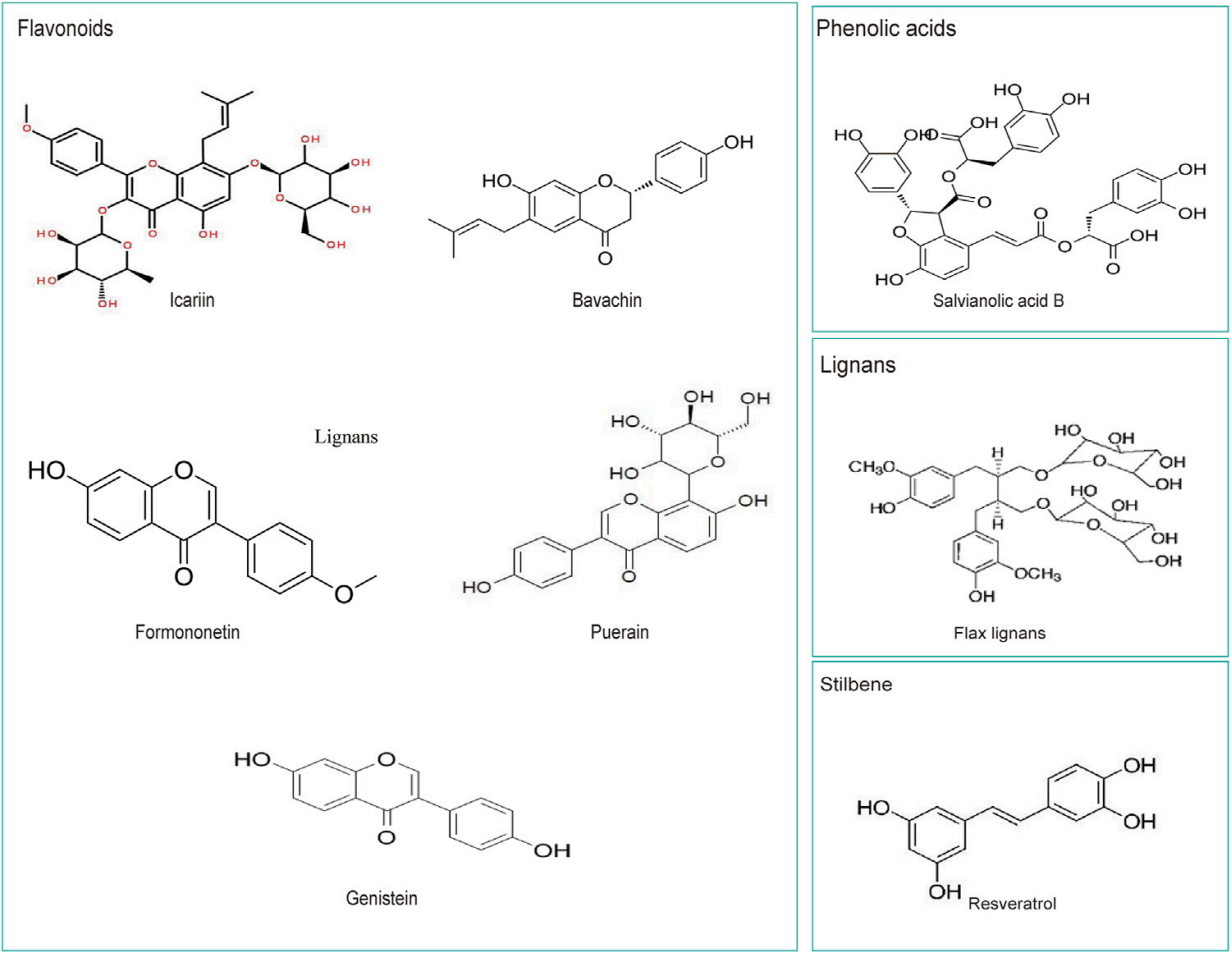
The chemical structures of several widely studied polyphenols in Chinese herbal medicine.

**FIGURE 3:**
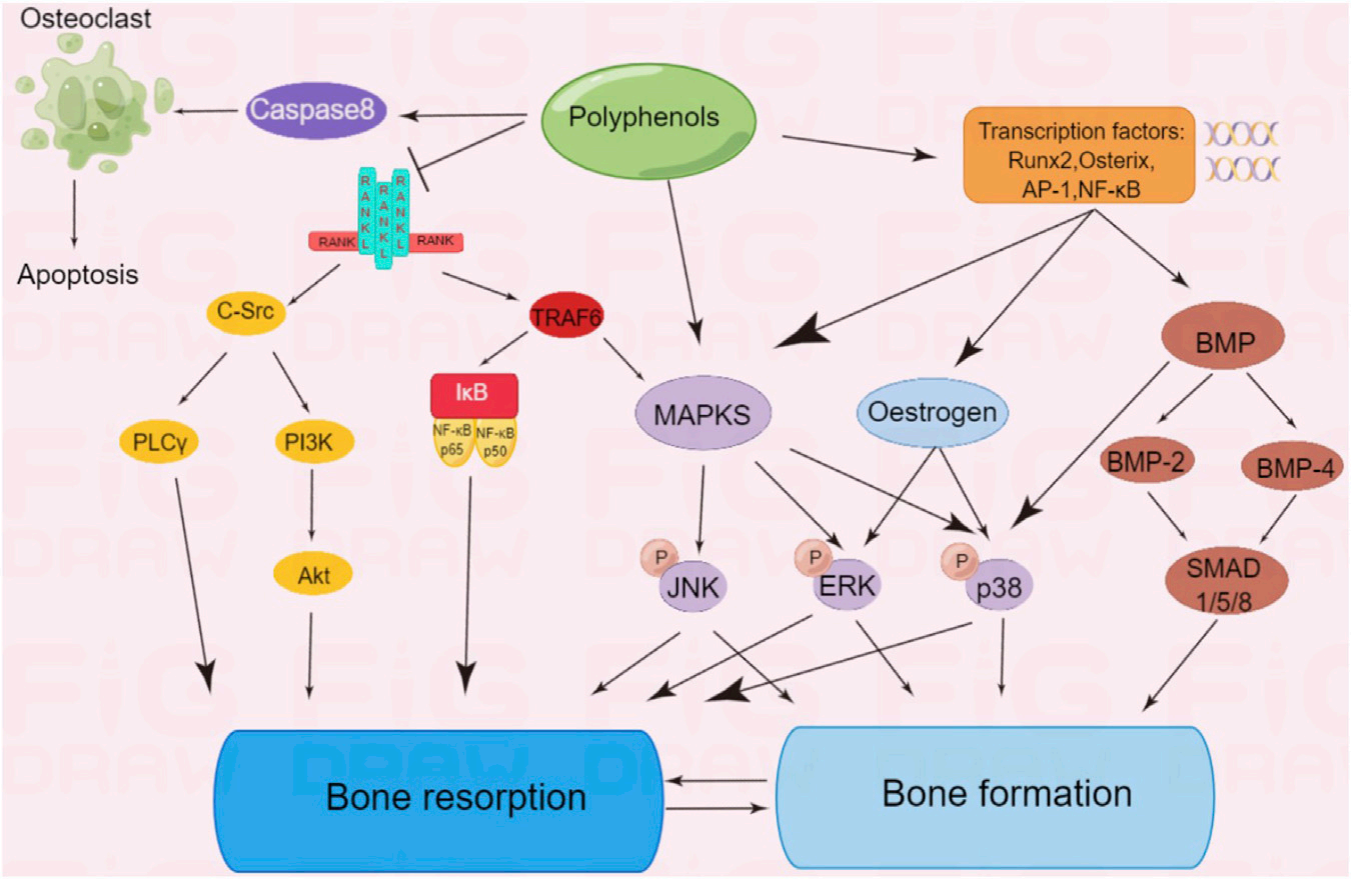
Polyphenols regulate multiple signaling pathways of bone formation or bone resorption.

In this paper, the research progress of the specific mechanism of polyphenol compounds on bone formation and bone absorption is reviewed. This paper provides a theoretical basis for the basic research of polyphenol compounds on bone formation and bone absorption (Figures 1–3). In addition to the text of the polyphenols, we have summarized the main traditional Chinese medicine of polyphenols components (Table 1).

**TABLE 1:** List of Chinese herbal polyphenols connected with the mechanism of bone formation or resorption.

Traditional Chinese medicine	Polyphenols	Mechanism of action	References
Glycyrrhiza uralensis	Glabridin	Glabridin shows slightly positive effect on osteoporotically changed bone tissue. And glabridin inhibiting RANKL-induced activation of signaling molecules and subsequent transcription factors in osteoclast precursors	Kim et al. (2012); Klasik-Ciszewska et al. (2016)
Fisch			
Glycyrrhiza uralensis	Isoliquiritigenin (ILQ)	ILQ reduces bone resorption *in vivo* and osteoclast differentiation *in vitro*, by mechanisms likely differing from actions of ovarian hormones.In addition, ISL directly reduced RANKL-RANK-TRAF6 singling pathway induced osteoclastogenesis	Zhu et al. (2012); Ji et al. (2018); Joyce et al. (2022)
Fisch			
Curcuma longa L	Curcumin	Curcumin Modulates the Crosstalk Between Macrophages and Bone Mesenchymal Stem Cells to Ameliorate Osteogenesis. And curcumin enhanced the BMSC function for the proliferation and migration of articular chondrocytes, and anabolic gene expression of extracellular matrix in articular chondrocytes *in vitro*, and the generation of articular cartilage *in vivo*.And Curcumin reduced apoptosis and promoted osteogenesis under oxidative stress	Yang et al. (2020); Chen et al. (2021); Tan et al. (2021); Zhang et al. (2021a)
Taxillus sutchuenensis (Lecomte)Danser	Quercetin	Quercetin was shown to inhibit RANKL-mediated osteoclastogenesis, osteoblast apoptosis, oxidative stress and inflammatory response while promoting osteogenesis, angiogenesis, antioxidant expression, adipocyte apoptosis and osteoclast apoptosis. The possible underlying mechanisms involved are regulation of Wnt, NF-κB, Nrf2, SMAD-dependent, and intrinsic and extrinsic apoptotic pathways	Tsuji et al., 2009; Mariee et al., 2012; Xing et al., 2017; Fayed et al., 2019; Xu et al., 2019; Nani et al., 2021
Tripterygiu m wilfordii < Hook. F	Celastrol	Celastrol could regulate BM-MSCs fate and bone-fat balance in OP and skeletal aging by stimulating PGC-1α. In addition, Celastrol inhibits glucocorticoid-induced osteoporosis in rat *via* the PI3K/AKT and Wnt signaling pathways. And Celastrol Attenuates RANKL-Induced Osteoclastogenesis *in vitro*	Xi et al., 2018; Li et al., 2020b; Xu et al., 2021
Zingiber officinale Roscoe	6-Gingerol	6-Gingerol Inhibits Inflammation-Associated Osteoclast Differentiation *via* Reduction of Prostaglandin E₂ Levels. And 6-Gingerol-stimulated osteoclast differentiation of bone marrow macrophages	Khan et al., 2012; Hwang et al., 2018; Zang et al., 2021
Lycium ruthenicum Murr	Anthocyanidin	Anthocyanins display their beneficial role on bone formation, including upregulating the osteoblastic genes, promoting the proliferation of osteoblasts and enhancing the mineral nodule formation	Li et al., 2017; Melough et al., 2017; Karunarathne et al., 2021; Liu et al., 2021
Rhodiola rosea L	Salidroside	Salidroside improves bone histomorphology and prevents bone loss rats by regulating the OPG/RANKL Ratio, the HIF-1α/VEGF signalling pathway, the Wnt/β-catenin signaling pathway	(Zheng et al., 2018; Guo et al., 2020; Li et al., 2021b
Crocus sativus L	Crocin	Anti-apoptotic effects, as well as osteoclast inhibition effects of crocin, have suggested it as a natural substance to treat osteoporosis and degenerative disease of bone and cartilage	Cao et al., 2014; Fu et al., 2017; Algandaby, (2019)
Reynoutria japonica Houtt	Polydatin	Polydatin improves osteogenic differentiation of human bone mesenchymal stem cells *via* BMP2-Wnt/β-catenin signaling pathway. In addition, Polydatin alleviates osteoporosis by enhancing the osteogenic differentiation of osteoblasts	Shen et al., 2020; Yuan et al., 2022
GALLA CHINENSIS	Epigallocatechin gallate (EGCG)	EGCG repressed new bone formation through Wnt/β-Catenin/COX-2 pathway. In addition, it may enhance bone defect healing *via* at least partly by the *de novo* bone formation of BMP-2	Lin et al., 2019; Zhang et al., 2021b
Davallia mariesii Moore ex Bak	Eriodictyol	Eriodicyol inhibits osteoclast differentiation and ovariectomy-induced bone loss *in vivo*. In addition, it Inhibits RANKL-Induced Osteoclast Formation and Function *Via* Inhibition of NFATc1 Activity	Lee et al., 2015; Song et al., 2016
Davallia mariesii Moore ex Bak	Naringenin	Naringenin promotes SDF-1/CXCR4 signaling pathway in BMSCs osteogenic differentiation. And naringenin is a Potential Anabolic Treatment for Bone Loss by Modulating Osteogenesis, Osteoclastogenesis, and Macrophage Polarization	Wang et al., 2021; Zhou et al., 2022

### 2.1 Flavonoids

#### 2.1.1 Icariin

Icariin.

Icariin (ICA) is 8-isopentenyl flavonoid glycoside and is the most abundant flavonoid active ingredient in epimedium. Bone marrow stromal cells (BMSCs) are stem cells isolated from adult bone marrow that have the ability to differentiate into osteoblasts, chondrocytes, adipocytes, and myoblasts. Epimedium has the ability to promote bone formation and can promote the proliferation of bone marrow mesenchymal stem cells and the differentiation of osteoblasts. At the level of the epigenetic regulation mechanism, ICA can regulate the homeostasis between osteogenic and adipogenic differentiation of mesenchymal stem cells (MSCs) through ABCB1 promoter demethylation (Sun et al., 2015). Similarly, it can also conduct epigenetic modification through miRNA. For example, studies have shown that ICA regulates the levels of Mir-23a-3p and Mir-335–5p and regulates the downstream pathway, thus affecting the osteogenic differentiation of BMSCs (Zhang et al., 2021; Teng et al., 2022). In addition, up-regulating the expression of Mir-335–5p and inhibiting phosphatase and tensin homolog deleted on chromosome ten (PTEN) can improve the susceptibility of osteoporosis (OP), thus providing new strategies for the prevention and treatment of OP (Teng et al., 2022). ICA can promote osteogenic differentiation by regulating the BMP/Smads pathway, the BMA1-BMP2 signaling pathway, and the BMP-2/Smad 5/Runx two and WNT/β-catenin pathways in BMSCs (Gao et al., 2021; Zhang et al., 2021; Jiao et al., 2022). Epimedium promotes the migration of bone marrow mesenchymal stem cells *in vitro* and *in vivo* through the MAPK signaling pathway (Jiao et al., 2018). In addition, ICA can promote the *in-situ* proliferation and osteogenic differentiation of bone marrow mesenchymal stem cells, thus improving the curative effect of bone marrow mesenchymal stem cell transplantation in the treatment of OP (Gao et al., 2021).

ICA can promote the differentiation of osteoblasts and increase bone mineral density. Bone formation primarily promotes the synthesis and mineralization of the bone matrix through the proliferation and differentiation of osteoblasts that play important roles in bone formation and osteoporosis. ICA has an estrogen-like pharmacological activity that can stimulate the differentiation and mineralization of osteoblasts, regulate the differentiation of osteoclasts, inhibit the adipogenic trans-differentiation of osteoblasts, and increase the number of osteoblasts differentiated into mature osteoblasts through the ER-mediated pathway (Zhang et al., 2016). *In vivo*, icariin increases the peak bone mass of rats during the growing period. Osteoblasts respond to icariin through the activation of cAMP/PKA/CREB signals. After the cAMP/PKA/CREB signal was blocked, icariin-induced osteogenesis was inhibited. These results further support that icariin promotes bone formation through the activation of the cAMP/PKA/CREB pathway (Shi et al., 2017). Icariin can also improve OP by regulating the balance of the EphB 4/ephrin-B2 pathway (Huang et al., 2020). Interestingly, ICA can also prevent the iron overload induced reduction of Runx2, alkaline phosphatase, and osteopontin expression, thereby inhibiting iron-induced osteoblast apoptosis and promoting bone formation (Jing et al., 2019). In addition, some studies have shown that icariin might exert an osteoprotective effect by maintaining osteocyte viability and thereby regulating bone remodeling (Feng et al., 2013; Ho et al., 2018; Park et al., 2019)

ICA inhibits the formation of osteoclasts, and ICA inhibits the differentiation of pre-osteoclasts to osteoclasts. It also inhibits the expression of various genes involved in osteoclast formation and bone resorption (Zhang S. et al., 2017). Studies have shown that icariin can block osteoclast formation induced by MCF seven and MDA-MB-231 breast cancer cells by inhibiting the activation of NF-κ B. In addition, icariin inhibits the expression of TRAF-6 stimulated by RANKL and then inhibits ERK phosphorylation, but it has no effect on the activation of p 38, JNK, and Akt (Kim et al., 2018). In addition, ICA can also prevent inflammatory bone loss. ICA inhibits the LPS-induced osteoclast formation process by inhibiting the activation of the p38 and JNK pathways (Hsieh et al., 2011).

In summary, ICA can prevent and treat osteoporosis by improving bone metabolism, promoting the differentiation of bone marrow mesenchymal stem cells, stimulating osteoblasts, and inhibiting osteoclast activity.

#### 2.1.2 Bavachin

Bavachin (BA) is the extract of the Chinese medicine *Psoralea corylifolia*. BA may stimulate bone formation or have potential anti-osteoporosis activity (Wang et al., 2001). BA can obviously stimulate cell proliferation and promote the differentiation of osteoblasts. This function may be related to its characteristic structure, that is, the isoprene side chains connected in each of its molecular skeletons (Li et al., 2014). It has been shown that BA can reduce bone turnover by decreasing serum alkaline phosphatase, serum carboxy-terminal collagen crosslinks (CTX) levels, and the urine deoxypyridinoline (u-DPD)/creatinine ratio, and preventing OVX-induced urinary calcium and phosphorus excretion. Similarly, BA can reduce the contents of gamma-aminobutyric acid (GABA) and GABABRI in the femur, increase the bone mineral density, and reduce urinary calcium excretion, thus achieving the purpose of preventing and treating osteoporosis *in vivo* (Zhu et al., 2015).

BA may inhibit osteoclast differentiation through the NF-κB signaling pathway and the MAPK signaling pathway *in vitro* (Wei et al., 2022). BA treatment can inhibit osteoclast function and promote the up-regulation and down-regulation of the osteoclast marker gene, RANKL, and the osteoblast marker gene, OPG. Serum aminoterminal propeptide of type I procollagen (PINP) is widely considered as a biomarker for evaluating osteoblast activity (Hale et al., 2007). It was found that BA significantly improved the level of serum PINP. These results indicated that BA not only has estrogen-like effects, but also has beneficial effects on the function of osteoblasts. BA can prevent OVX-induced bone loss, but it does not affect uterine estrogen. This type of bone protection makes this a promising alternative to treat postmenopausal osteoporosis (PMOP) safely and effectively (Weng et al., 2015).

BA can achieve the purpose of anti-osteoporosis through a delicate balance of bone formation and bone resorption.

#### 2.1.3 Formononetin

Formononetin (FO) is one of the primary isoflavones extracted from *Astragalus membranaceus*. Studies have shown that it can stimulate the formation of osteoblasts, thus increasing bone mass and improving the microstructure of bone. FO can regulate the expression of RANKL and OPG at the mRNA level, as well as related markers of osteogenic differentiation, thus promoting the mineralization potential of osteoblasts (Zaklos-Szyda et al., 2020). In addition, FO promoted bone regeneration in a mouse model of cortical bone defect in a manner similar to PTH and upregulated the expression of the predominant runt-related transcription factor 2 and osteocalcin (Singh et al., 2017). The research results showed that FO inhibited fat formation through the AMPK/β-catenin signal transduction pathway, thus improving the inverse relationship between osteoblasts and adipocytes and preventing obesity and bone loss induced by high-fat diets (Gautam et al., 2017).

FO can inhibit the activation of osteoclasts and plays an important role. Studies have shown that FO can inhibit the proliferation and differentiation of primary bone marrow mononuclear macrophages into osteoclasts and down-regulate the expression of proteins and genes related to the bone resorption function of osteoclasts, and this may be one of the mechanisms of FO in preventing and treating destruction and collapse in femoral head necrosis (Hong et al., 2020). FO attenuates osteoclast differentiation and calcium loss by regulating the transcription factor, AP-1, in type I diabetic mice, and it is expected to be a prospective drug for the treatment of osteoporosis (Jing et al., 2022).

The immunomodulatory activity of formononetin can prevent OVX-induced bone loss. In addition, the generation of osteoclasts and apoptosis of osteoblasts induced by IL-17 are inhibited (Mansoori et al., 2016). FO can reduce the production of osteoclasts by inhibiting the activation of NF-κ B, c-fos, and nuclear factors that activate the cytoplasmic one signal pathway induced by RANKL in T cells (Huh et al., 2014). FO also has estrogen-like effects that can inhibit bone loss caused by estrogen deficiency after menopause and improve the activity of alkaline phosphatase in OVX rats *in vivo* and *in vitro* (Ha et al., 2010).

In a word, FO can stimulate the formation of osteoblasts and inhibit the activation of osteoclasts.

#### 2.1.4 Puerarin

Pueraria, originating from China, has a long history and is one of the most commonly used Chinese medicines in Asia. Due to its high isoflavone content, it has been widely used as a natural alternative to hormone replacement therapy for postmenopausal symptoms (Lee et al., 2021). Puerarin (PUE) is an isoflavone isolated from the pueraria root that is widely distributed in several organs related to aging, such as the hippocampus, femur, tibia, and mammary gland, after oral administration (Anukunwithaya et al., 2018). In addition to anti-inflammatory, antioxidant, and anti-diabetic effects (Xiao et al., 2011; Chen et al., 2018; Jeon et al., 2020), PUE also plays an important role in bone diseases such as OP. PUE can promote bone formation by influencing the expression of osteogenic related genes and promoting the formation of a mineralized matrix. PUE can significantly enhance alkaline phosphatase activity, mineralized matrix generation, and osteoblast-related protein expression levels. In addition, microCT imaging measurements demonstrated that PUE significantly promoted new bone formation (Yang et al., 2022). At the level of epigenetic modification, PUE regulates transcriptional expression related to bone formation through microRNA (Shan et al., 2018; Zhou et al., 2020). For example, PUE can regulate the up-regulation of Mir-155–3p to promote BMSCs differentiation and bone formation and increase bone mass in bone grafted rats.

Studies have shown that PUE also has a regulatory effect on bone resorption. PUE can down-regulate the mRNA levels of osteoclast marker genes CTR, CATH-K, NFATc1 and c-fos, indicating that PUE inhibits osteoclast cell function *in vitro* (Yang et al., 2019). *In vitro*, PUE attenuated bone resorption without impairing osteoclast viability and significantly prevented OVX-induced bone loss by inhibiting bone resorption without altering bone formation (Qiu et al., 2022). Furthermore, PUE inhibites RANKL-induced osteoclast activation, the bone resorption capacity, and F-actin ring formation *in vitro* with an increase in the PUE concentration (Yang et al., 2019). PUE may play a protective role in osteoclast-related osteolytic diseases. *In vitro*, PUE prevented RANCL-induced osteoclast differentiation, bone resorption, and F-actin ring formation, reduced phosphorylation of p65, and prevented P65 translocation from the cytoplasm to the nucleus in a concentration-dependent manner. PUE also decreased the expression of osteoclast specific factors (Tang et al., 2020). *In vivo* experiments, PUE significantly inhibited bone resorption mediated wear particles in a skull bone resorption model (Yang et al., 2019).

PUE can also prevent cell apoptosis through the HDAC1/HDAC3 signaling pathway and regulate the expression of HIF-1α, TIMP-3, and Bcl-2, thus playing an anti-osteoporosis role (Guo et al., 2019; Waqas et al., 2020). For osteoporosis, it also increases bone mass and inhibits osteoclast formation (Yang et al., 2018; Xiao et al., 2020). By enhancing osteogenesis and promoting bone formation, PUE also improves OVX-induced osteoporosis and lipid metabolism by regulating phospholipid metabolism and polyunsaturated fatty acid biosynthesis, thereby reducing adipogenic differentiation. In addition, activation of the Wnt pathway and inhibition of the PPARγ pathway promote adipogenesis in osteogenic differentiation of inactivated rat bone marrow mesenchymal stem cells (Li et al., 2022). For osteoporosis caused by postmenopausal estrogen deficiency, the results of a clinical trial showed that PUE was well tolerated for the short-term treatment of mild to severe menopausal symptoms in women. Kudzu root extract may benefit bone and cartilage health and may be a promising natural alternative to existing treatments for menopausal symptoms (Bihlet et al., 2021).

Recent studies have found that the microbiota plays an important role in regulating the skeletal microenvironment, thereby triggering anti-osteoporosis effects. Furthermore, intestinal microbiota can participate in the process of osteoporosis by inducing inflammatory reactions and changes in the autoimmune system. PUE treatment can improve the bone microenvironment and inhibit OVX-induced osteoporosis by regulating the release of short chain fatty acids (SCFAs) from intestinal flora and repairing the intestinal mucosal integrity (Li B. et al., 2020). In addition, serum pharmacokinetics suggest that pueraria root extract may undergo enterohepatic circulation (Mun et al., 2009).

#### 2.1.5 Genistein

Genistein is a natural isoflavone compound found in legumes and dentate plants. It is a phytoestrogen that makes up more than 60% of soy isoflavones (Nazari-Khanamiri and Ghasemnejad-Berenji, 2021). Its pharmacological properties make it a potential drug for treating a variety of conditions including postmenopausal symptoms, cancer, and bone, brain, and heart disease (Nazari-Khanamiri and Ghasemnejad-Berenji, 2021).

It is well known that genistein has been shown to stimulate bone formation by osteoblasts and inhibit bone resorption by osteoclasts, thereby increasing bone mass (Yamaguchi, 2012). Genistein improves bone healing by triggering the estrogen receptor α-mediated expression of osteogenesis-related genes and maturation of osteoblasts. The inhibition of ER expression was shown to immediately reduce the genistein-induced enhancement of mitochondrial energy production and osteoblast activation (Wu et al., 2020). Additionally, studies have shown that genistein promotes osteoblast differentiation and maturation by activating the ER, p38MAPK-Runx2, and NO/cGMP pathways and by inducing the osteoclastogenesis inhibitor, osteoprotegerin (OPG), and blocking the NF-κB signaling pathway, inhibiting osteoclast formation and bone resorption (Ming et al., 2013).

At the level of epigenetic modification, genistein counteracts NF-κB-induced osteoclast generation and downstream signaling by directly regulating the transcription of histone methyltransferases EzH2 and EzH1 (Kushwaha et al., 2022). Furthermore, there are clinical trials showing that supplementation with the dietary phytoestrogen genistein may be as effective as hormone replacement therapy in reducing bone loss associated with menopause without the associated bone loss side effects (Cotter and Cashman, 2003; Sansai et al., 2020). In this study, to improve the bioavailability of genistein and reduce its side effects, the nanofied formulation of genistein with Vitamin D was invented to enhance the therapeutic efficacy of the osteoporosis model *in vitro* and improve alkaline phosphatase activity and multinucleated giant cell formation (Kushwaha et al., 2022). If the bioavailability of genistein is improved, its future market development potential is huge.

### 2.2 Phenolic acids

#### 2.2.1 Salvia B

*Salvia miltiorrhiza* Bunge, also known as *Salvia miltiorhiza* Bunge, is often used in traditional Chinese medicine (TCM) in combination with other traditional Chinese medicines to treat bone diseases. Salvianolic acid B (Sal B) is a water-soluble phenolic compound isolated from *Salvia miltiorrhiza* Bunge in which the active ingredient in the water-soluble phenolic compound is greater than 50% (Cao et al., 2012). As a polyphenolic acid compound with seven phenolic hydroxyl groups, Sal B is one of the strongest natural antioxidants, and it is metabolized into salvianol *in vivo* (Chen et al., 2013; Tang et al., 2014).

It is worth noting that Sal B can also act on osteoblasts and induce bone marrow-derived mesenchymal stem cells to become osteoblasts. Studies have shown that Sal B and *Salvia miltiorrhiza* can induce osteogenic differentiation of rat bone marrow stromal cells by up-regulating the nitric oxide pathway (Zhang X. et al., 2017). In addition, Sal B can protect osteoblasts treated with prednisolone acetate by stimulating the activity of osteoblasts and the expression of genes related to bone formation and differentiation. It can also increase the alkaline phosphatase (ALP) activity in osteoblasts and stimulate the expression of ALP, which is inhibited by prednisolone acetate, and up-regulate the expression of Runx2, Osx, OCN, IGF-I, Col-I, HO-I, mRNA, and protein expression (Qiao et al., 2019). For the first time, studies have shown that Sal B can target TAZ to promote osteogenesis and reduce adipogenesis by activating MEK-ERK signaling pathway, which provides evidence that Sal B can be used as a potential therapeutic agent for the management of bone diseases (Wang et al., 2019). Sal B can also play a cytoprotective role to inhibit the apoptosis of BMSCs by regulating H2O2-mediated ROS/MEK/ERK1/2 pathway (Lu et al., 2010). This indicated that Sal B had a protective effect on osteoblasts by stimulating osteoblast activity and the expression of genes related to bone formation and differentiation.

### 2.3 Lignans

#### 2.3.1 Flax lignans

Flax lignans are phytonutrient extract of *Linum usitatissimum* L. Chemically, the C6-OH of the glucose of flax lignans is esterified to the carboxylic acid of hydroxymethylglutaric acid (Imran et al., 2015). Flax lignans in combination with low-dose estrogen treatment maximally prevents bone loss induced by oophorectomy (Sacco et al., 2009). However, its use alone has no effect on the bone mineral density content, and a clinical study showed no statistically significant difference in bone turnover markers between the treatment group and the placebo group (Alcorn et al., 2017). Flax lignans have no negative effects on bone strength and bone health in aged rats (Ward et al., 2001a). These studies indicate that supplementation with flaxseed may contribute to improving the bone properties of osteoporosis, and these predominantly protective effects may be attributed to flaxseed oil (predominantly ALA), not to the fractions of flax lignans (Ward et al., 2001b; Lucas et al., 2002; Cohen and Ward, 2005). Flax Lignans are characterized by anti-inflammatory, antioxidant, and neuroprotective properties (Watanabe et al., 2020; Asad et al., 2021; Wu et al., 2021).

### 2.4 Stilbenes

#### 2.4.1 Resveratrol

Resveratrol (RES), a non-flavonoid polyphenolic organic compound and is a bioactive component in Rhizoma Polygoni Cuspidati. It is easily absorbed after oral administration and is excreted through the urine and feces after metabolism. A large number of experimental studies have shown that RES has antioxidant, anti-inflammatory, anti-cancer, and cardiovascular and cerebrovascular protection effects (Biswas et al., 2020; Thaung Zaw et al., 2021; De Luca et al., 2022; Dzator et al., 2022; Mahjabeen et al., 2022).

Previous studies have shown that RES also plays an important role in protecting and promoting early bone metabolism and differentiation through a mechanism similar to genistein that promotes osteoblast-mediated bone formation and inhibits osteoclast-stimulated bone formation (Tou, 2015). RES increased the serum OPG, femoral SIRTI, and β-catenin expression and significantly decreased the NF-κB ligand receptor activator (RANKL) by stimulating SIRT1 expression and Wnt/β-catenin signaling. Finally, the bone mass of the femur increased and the bone mineral density significantly increased (Wang et al., 2022). In addition, RES can also protect bone cells from some physicochemical damage. For example, studies have shown that RES pretreatment for 30 min can significantly prevent cadmium-induced apoptosis and attenuate ERK1/2 and JNK signaling by regulating ERK1/2 and JNK signaling. It also produces cadmium-induced inhibition of osteogenic differentiation (Mei et al., 2021).

The aging of mesenchymal stem cells (MSCs) and the associated decline of osteogenic function lead to the disruption of the balance between bone formation and resorption, which is the key pathogenesis of osteoporosis during aging. Recent data has shown that RES can improve the osteogenic differentiation of senescent BMMSCs, and long-term intermittent applications can enhance bone formation and compensate for bone loss. The specific mechanism is that RES up-regulates Mitofilin, promotes the transcription of mitochondrial autonomous genes, and restores cell metabolism through mitochondrial function (Lv et al., 2018). Mitofilin, also known as the mitochondrial inner membrane protein (IMT) or Mic60, is a core component of the mitochondrial contact site and crista tissue system (MICOS) (Li et al., 2016; Tarasenko et al., 2017). Mitofilin is indispensable for mitochondrial homeostasis and osteogenesis in bone marrow mesenchymal stem cells (Chen et al., 2008; Pietilä et al., 2010). Mitofilin deficiency leads to aging and bone loss in BMMSC (George et al., 2011; Sahin et al., 2011).

Relevant clinical trials have also proved the efficacy and safety of RES. A clinical study conducted at the Aarhus University Hospital showed that high-dose RSE supplements increased bone mineral density (BMD) and bone alkaline phosphatase in obese men, with positive effects on bones (Ornstrup et al., 2014). A 24-month RES (RESHAW) trial of healthy ageing in women showed that regular 75 mg resveratrol supplementation twice daily had the potential to slow bone loss in the lumbar spine and femoral neck, which is common at fracture sites in postmenopausal women without significant osteoporosis (Wong et al., 2020). However, a systematic review and meta-analysis showed that RES supplements did not show any significant effect on BMD or serum bone markers (Li Q. et al., 2021). Therefore, further research utilizing better organized multicenter randomized trials is necessary so that physicians can provide more advice for clinical decision-making.

## 3 Conclusion

The incidence of bone metabolic diseases has been increasing annually. The imbalance of bone formation and absorption is an important mechanism of bone metabolism related diseases. Chinese herbal medicine has been used for thousands of years, among which polyphenols are an important active ingredient. This paper reviewed how polyphenols in Chinese herbal medicine can help bone reconstruction and improve bone metabolism by affecting the balance between bone formation and bone absorption. Generally speaking, regarding the beneficial effects of polyphenols in bone metabolic diseases, due to the lack of multi-center randomized trials in polyphenols in this field, it is considered necessary to conduct human trials, and further research can be conducted in this research field.
